# Pediatric tooth extractions under sedoanalgesia

**DOI:** 10.12669/pjms.325.10643

**Published:** 2016

**Authors:** Ayse Hande Arpaci, Berrin Isik

**Affiliations:** 1 Dr. Ayse Hande Arpaci, MD. Assistant Professor, Anaesthesiology Reanimation Specialist Oral & Maxillofacial Department, Faculty of Dentistry, Ankara University, Besevler, Ankara, Turkey; 2 Dr. Berrin Isik, MD. Professor, Anaesthesiology Reanimation Specialist, Faculty of Medicine Dept. of Anesthesiology and Reanimation, Gazi University, Besevler, Ankara, Turkey

**Keywords:** Dental treatment, Postoperative complications, Sedation

## Abstract

**Objective:**

The present study aims to evaluate intravenous ketamine and inhalation sedation in children, their unwanted side-effects and surgeon satisfaction.

**Methods:**

In this study, data of 922 children aged between 1-18 who underwent tooth extraction under sedoanalgesia in our department between September 2015-January 2016 were gathered and anesthesia approaches, unwanted side effects and surgical satisfaction was investigated. Postoperative recovery emergence agitation or delirium was evaluated with Watcha Behavior Scale (WBS).

**Results:**

Patients were grouped and compared according to acceptance of intravenous line placement (Group-1) or not (Group- 2). Group 1 received intravenous ketamine anesthesia (n=822), Group 2 received inhalation anesthesia with sevoflurane (n=100). Number of patients, age, weight and gender was significantly different in two groups. When side effects were investigated nausea was observed in 30 patients (3.6%), skin rashes were observed in 26 patients (3.2%) in Group-1 while skin rashes were observed in one patient (1%) in Group 2. 95% of surgeons reported intravenous anesthesia, 18% of surgeons reported inhalation anesthesia to be the anesthesia of choice. Emergence of postoperative recovery agitation (WBS≥3) was observed more frequent in Group 2 (p<0.05) than Group 1.

**Conclusion:**

Ketamine, which has analgesic, hypnotic and amnestic effects and which does not alter pharyngeal and laryngeal reflexes thus minimizes aspiration possibility, is a safe and effective anesthetic agent for tooth extractions of the pediatric population under sedoanalgesia.

## INTRODUCTION

Fear and anxiety in dental practice complicates the procedure or even sometimes it becomes impossible.^1^ Children with dental anxiety have been reported to have more caries counts and discontinue follow-ups.^2^ and to have more emergency administrations in order to treat dental pain and infections.^3^ It is the dentist’s duty to administer dental treatment to the child by using behavioral methods however in some children these behavioral and psycho pedagogic methods are difficult.^4^ Furthermore mental motor problems creating cooperation difficulty in children make sedation or general anesthesia inevitable.

In these situations, sedation is preferred because the patient is cooperative and the physiological reflexes are protected which is known to outclass general anesthesia in which the patient is uncooperative and the airway needs protection.^5^

Presented in 1999 and revised in 2004, the American Society of Anesthesiology (ASA) classified sedation procedures as minimal sedation (anxiolysis), medium sedation/analgesia (conscious sedation), deep sedation/analgesia and general anesthesia.^6^ Minimal and medium sedation/analgesia or sedoanalgesia is commonly preferred in dental practice because the physiological reflexes are protected, patient is cooperative and postoperative recovery is fast.

Furthermore patient beds are less occupied, operation room is more abundant, safety and patient comfort and satisfaction is increased.^7^ On the other hand, sedation depth may be increased in order to obtain deep sedation even general anesthesia and cardiorespiratory depression may develop. Even though appropriate anesthetic agents are used with recommended doses, unwanted effects of sedation can be seen^8^,^9^ and it is important to present how often these side effects occur and which risk factors trigger them. For these reasons, in this study, we aimed to share our experience, anesthetic agent chosen, associated postoperative complications, their frequencies and risk factors in pediatric patients who underwent tooth extraction under sedoanalgesia.

## METHODS

Data of children aged 1-18 who admitted to Ankara University, Faculty of Dentistry, Oral and Maxillofacial Surgery Department for tooth extraction between September 2015 - January 2016 were included in the study. This study protocol was approved by the ethic committee. Patients were directed to sedoanalgesia after unsuccessful tooth extraction attempts with local anesthesia or when they were uncooperative (mentally retarded etc.). Data were obtained from anesthesia records. Patients with incomplete data, with operation time longer than 20 minutes, who had active upper respiratory airway infection, lung infection or inflammatory disease, previous history of dental extraction and patients who did not met the 6 hours famine criteria did not receive sedoanalgesia thus were excluded from the study. Patients included in the study all met the 6 hours of famine criteria and patients and parents were informed about sedoanalgesia and tooth extraction. Before procedures all children were evaluated and physical examinations were made, further laboratory examination and consultations were demanded in cases of need.

Procedures took place in our department’s operation room and electrocardiography (EKG), heart beat rate (HBR), mean non-invasive arterial pressure (MNAP) and peripheral oxygen saturation (SpO_2_) was monitored (Nihon Kohden Bedside Monitor® model BSM-4113K-Japan) for all patients. Patients who enabled catheter insertion were administered intravenous (IV) 1-2mg kg^-1^ Ketamine (Ketalar®, Pfizer, New York, USA). If the patients need additional doses of ketamine for maintaining sedation level, 0.25-0.5 mg/kg iv ketamine were added.

Patients who did not allow intravenous line insertion were administered %50/50 O_2_/N_2_O containing 6% sevoflurane via mask. Sedation level was aimed to be Modified Ramsey Sedation Scale (MRSS) = 4.

Patients who reached MRSS=4 were oxygenized 4L/minute, with nasal cannula and administered local infiltration anesthesia (Ultracain®). Patients who were administered inhalation anesthesia (IA) were oxygenized 4L/minute when MRSS 4 was reached and other inhalation agents were ceased.

Patient age, body weight, gender, ASA risk class, additional systemic diseases, anesthetic agents used during procedure, number of extracted teeth, side effects observed during and after procedure and surgeon satisfaction were retrospectively evaluated using archived data. Postoperative recovery agitation or delirium was evaluated with Watcha Behavior Scale (1:Calm, 2:Crying, but can be consoled, 3:Crying, cannot be consoled, 4:Agitated and thrashing around).

### Statistical Analysis

Statistical analysis was made using SPSS 12.0 computer program using the below stated tests. Statistical analysis data were presented as Mean±Standart Deviation (SD). Statistical significance was accepted p<0.05 in all analysis. Kolmogorov-Smirnov test was applied to all measurable parameters to identify normal or abnormal distribution. To identify difference between groups in normally distributed data, Student-t test was used in independent groups. Gender, ASA, additional systemic diseases, side effects and surgeon satisfaction evaluation was made with Chi-square and Fisher’s exact Chi-square tests.

## RESULTS

As result of data evaluation it was detected that 922 patients under age of 18 underwent sedation/analgesia for tooth extraction. Demographic information of patients is presented in Table-I.

**Table-I:** Demographic features of patients [Mean ± SD) (min-max), n(%)]

*Patient number*	*n=922*
Age (year)	6.04±2.91 (1-18)
Gender (M/F)	564 (61.2)/358 (38.8)
Weight (kg)	23.39±10.71 (6-82)
ASA (1/2)	822 (89.2)/100 (10.8)
Additional systemic disease (Not present /Present)	885 (96)/37 (4)

Patients were divided into two groups according to anesthesia type, Group-1 received IV ketamine anesthesia, Group- 2 received IA (sevoflurane). Age, weight and gender was significantly different in two groups. ASA and additional systemic diseases were similar in two groups (Table-II).

**Table-II:** ASA and additional systemic disease data, extracted tooth number and side effect data [Mean ± SD) (min-max), n(%)]

		*Group 1 (n=822)*	*Group 2 (n=100)*	*P*
Age (year)		6.23±2.91 (1-18)	4.50±2.36* (1-16)	<0.0001
Gender (M/F)		512 (62.3)/310 (37.7)	52 (52)/48 (48)*	0.046
Weight (kg)		23.83±10.95 (6-82)	19.74±7.66* (8-45)	<0.0001
ASA (1/2)		730 (88.8)/92 (11.2)	92 (92)/8 (8)	0.332
Additional systemic disease (Not present /Present)		787 (95.7)/35 (4.3)	98 (98)/2 (2)	0.414
Anesthesia		822 (89.2)	100 (10.8)	
Extracted Tooth Number		2.89±1.96 (1-13)	2.35±1.72* (1-9)	0.009
Side Effects	Not Present	766 (93.2)	99 (99)	0.011
	Nausea	30 (3.6)	0 (0)	
	Skin Rashes	26 (3.2)	1 (1)	
	Watcha Behavior Scale (n=1/2/3/4)	205/328/247/42	6/19/69/6	<0.001

* p<0.05: Compared to Group 1

Eight hundred twenty two patients (89.2%) included in the study were administered IV anesthesia (Group- 1), 100 patients (10.8%) were administered IA Group-2) (Table-II). Extracted tooth numbers were significantly higher in Group-1 compared to Group-2. When side effect data was evaluated, nausea was seen in 30 patients in Group 1 (3.6%), skin rashes were seen in 26 patients in Group 1 (3.2%) and 1 patient in Group 2 (1%), Number of the patients whose WBS scores (1/2/3/4) in Group 2 were 205/328/247/42 and 6/19/69/6 in Group 1 respectively. In spite of saliva secretion much more than Group 2 in Group 1, it was removed with suction continuously and it did not cause of serious problem.

Side effect occurrence rate was significantly higher in Group 1 compared with Group 2 (Table-II). Postoperative recovery emergence agitation (WBS≥3) was observed more frequently in Group 2 (p<0.05) than Group 1. When surgeon satisfaction was evaluated, 95.5% of surgeons favored for IV anesthesia and 18% of patients favored for IA. Surgeon satisfaction was significantly higher in Group 1 compared to Group 2 (Table-III).

**Table-III:** Surgeon satisfaction data [n(%)]

		*Group 1 (n=822)*	*Group 2 (n=100)*	*P*
Surgeon Satisfaction	Excellent	785 (95.5)	18 (18)	<0.0001
	Very good	37 (4.5)	71 (71)	
	Good	-	11 (11)	
	Medium	-	-	
	Bad	-	-	

## DISCUSSION

Dental anxiety of patients is a major problem affecting dental practice. Dental anxiety is influenced by various factors including age, gender, and socioeconomic conditions.^10^-^12^ Many studies have shown the inverse correlation between age and dental anxiety levels. Dental anxiety has shown to decrease by the age of 6-7 and that children can cope with the fears that cause dental anxiety more efficiently.^11^,^13^ Mean age of 922 patients who underwent tooth extractions under sedoanalgesia was in line with literature.

Mean age of patients in Group 1 was found to be significantly higher than patients in Group 2 (p<0.0001). The lower age group did not allow catheter insertion thus were administered IA and were included in Group 2. We are of the opinion that this is the main reason for the age difference between two groups.

When gender and dental anxiety levels are correlated, although contradictory, many studies state that males tend to have higher levels than females.^1^,^14^ In line, 61.2% of 922 patients included in our study were male. However other sociological factors contributing to this result may be present.^10^-^12^

A study conducted with patients with dental anxiety came to the conclusion that these patients had more caries counts, less teeth with fillings and more missing teeth, did not continue follow-ups regularly and had more emergency administrations in order to treat dental pain and infections.^1^-^3^ In our study minimum one and maximum 13 teeth extraction was noted. We associated this gross number of extraction indication to dental anxiety thus delayed dentist examinations.^15^

Dental anxiety is reported to be 3-43% in the pediatric population.^16^ When behavioral methods are insufficient in eliminating this condition, sedoanalgesia or general anesthesia is needed. Sedation in which the patient is cooperative and the physiological reflexes are protected is usually preferred over general anesthesia^5^ in the same way as our department. Like appropriate patient selection for ambulatory dental practice, short-term anesthetics, which have little side effects and provide fast recovery, are reported to decrease morbidity rate.^7^

Today, ketamine is the only known anesthetic agent with analgesic, hypnotic and amnestic effects, which also protects pharyngeal and laryngeal reflexes and does not evoke cardiovascular and respiratory depression. In anesthesia literature, ketamine is the anesthetic agent of choice for obtaining sedation in the pediatric population during dental procedures.^17^ It is an effective agent for painful and unpleasant diagnostic and therapeutic procedures in dental practice, which provides analgesia, sedation, anxiolysis, amnesia, and a short duration of motor control.^17^

The most commonly used inhalation agent used in dental practice is nitrous oxide. Although this agent has strong analgesic potency strength, it is a weak anesthetic agent and may be insufficient in patients with high anxiety levels. In our department, children who underwent examination in our pediatric clinic and did not allow examination, or who did not consent tooth extraction in the clinic after at least two attempts are directed to sedoanalgesia thus have high fear and anxiety levels. Therefore we aim MRSS level 4 in our sedation practice and give preference to sevoflurane, which has lower blood-gas solubility compared to other inhalation agents, has fast-paced effect, which ends fast and does not cause airway irritation.^16^-^18^ When 2400 closed cases in the USA were evaluated, it was observed that 238 of these cases were pediatric, which involved respiratory problems different from adults and was concluded that the mortality rate could be decreased with better monitorization.^9^,^19^ Another study evaluated 95 cases in hospitals and private practice and concluded that 78% of cases where pulse oximeter monitoring was not present, unwanted occurrence was associated with mortality or neurological deficits whereas this rate decreased to 28% in patients who were monitorized with pulse oximeter.^20^ In our practice we choose to monitor respiratory and circulatory system functions (EKG, HBR, MNAP and SpO_2_) and oxygenized the patients with nasal cannula in order to prevent these possible complications. We have not come across respiratory complications or any serious desaturation or severe emergency agitation or delirium during our practice. Patient MRSS levels were evaluated closely in order to prevent cardiorespiratory complications associated to deepening of anesthesia.

The main adverse effect is vomiting reported in approximately 10% of pediatric patients undergoing sedoanalgesia.^21^ Nausea after ketamine administration seems to be associated with increasing age.^21^ Ozkan et al.^22^ reported 12.1% nausea and 7.9% vomiting during their practice of elective surgery in the pediatric population. Salleeh et al.^23^ used ketamine in 179 pediatric emergency patients for painful and traumatic, diagnostic and therapeutic procedures and reported 60% of vomiting to be the most frequently observed side effect. In our study 30 patients in Group 1 (3.3%) had nausea in line with literature, but we did not come across vomiting. We are of the opinion that this result is related with our administration of antiemetic medication in children with nausea.

In literature a 3-year-old child is reported to develop skin rashes and anaphylactoid reaction with intramuscular ketamine administration.^24^ We are of the opinion that in our study, eritematous skin reactions, which regressed spontaneously in 26 patients (3.2%) in Group 1 were caused by fast injections and following acute increase in plasma histamine levels.

The emergence of agitation (EA) occurrence in children after sevoflurane anesthesia is common, with a reported incidence up to 80%. EA is characterized by restlessness, inconsolable crying, agitation, disorientation, delusion, hallucination and cognitive changes plus memory impairment. EA in children is generally short-lived with no after-effect, however, it is a troublesome phenomenon, because it can result in injury to the patient or damage to the surgical site, leads to dissatisfaction and anxiety for the parents, and requires extra nursing care with associated costs.^25^ In our research we determined EA in two groups. But EA was more comman in Group 2 than Group 1.

When surgeon satisfaction was evaluated, 95.5% of surgeons found IV anesthesia to be ‘Excellent’ while 18% of surgeons found IA to be ‘Excellent’. When satisfaction rate was compared there was a significant difference in Group 1 and Group 2. Surgeon’s thought on what brought this difference out was that they could start the procedure more quickly when IV anesthesia was administered and that the effect of anesthesia terminated more quickly (MRSS 2 level achieved), again gaining time for the surgeon. Other causes were that after being sent to the recovery room and after total recovery, children did not remember the procedure and did not cry when they saw the surgeon and that less gas was polluting the operating room.

## CONCLUSION

Dental anxiety in the early ages and high prevalence leads to the need of sedoanalgesia during dental procedures of these children. Ketamine is an effective and safe agent with analgesic, hypnotic and amnestic affects, does not alter pharyngeal and laryngeal reflexes and minimizes aspiration possibility during procedure. We feel that ketamine can be preferred to other agents for pediatric sedoanalgesia.
